# Suppression of mitochondrial respiration with auraptene inhibits the progression of renal cell carcinoma: involvement of HIF-1α degradation

**DOI:** 10.18632/oncotarget.5511

**Published:** 2015-10-12

**Authors:** Yunseon Jang, Jeongsu Han, Soo Jeong Kim, Jungim Kim, Min Joung Lee, Soyeon Jeong, Min Jeong Ryu, Kang-Sik Seo, Song-Yi Choi, Minho Shong, Kyu Lim, Jun Young Heo, Gi Ryang Kweon

**Affiliations:** ^1^ Department of Biochemistry, Chungnam National University School of Medicine, Daejeon, Republic of Korea, 301-747; ^2^ Research Institute for Medical Science, Chungnam National University School of Medicine, Daejeon, Republic of Korea, 301-747; ^3^ R&D center, KT&G Life Sciences; ^4^ Department of Pathology, Chungnam National University School of Medicine, Daejeon, Republic of Korea, 301-747; ^5^ Department of Internal Medicine, Chungnam National University School of Medicine, Daejeon, Republic of Korea, 301-747; ^6^ Brain research institute, Chungnam National University School of Medicine, Daejeon, Republic of Korea, 301-747

**Keywords:** auraptene, renal cell carcinoma, HIF-1α, mitochondrial respiration, eIF2α

## Abstract

Renal cell carcinoma (RCC) progression resulting from the uncontrolled migration and enhanced angiogenesis is an obstacle to effective therapeutic intervention. Tumor metabolism has distinctive feature called Warburg effect, which enhances the aerobic glycolysis rapidly supplying the energy for migration of tumor. To manipulate this metabolic change characteristic of aggressive tumors, we utilized the citrus extract, auraptene, known as a mitochondrial inhibitor, testing its anticancer effects against the RCC4 cell line. We found that auraptene impaired RCC4 cell motility through reduction of mitochondrial respiration and glycolytic pathway-related genes. It also strongly disrupted VEGF-induced angiogenesis *in vitro* and *in vivo*. Hypoxia-inducible factor 1a (HIF-1a), a key regulator of cancer metabolism, migration and angiogenesis that is stably expressed in RCCs by virtue of a genetic mutation in the von Hippel–Lindau (VHL) tumor-suppressor protein, was impeded by auraptene, which blocked HIF-1a translation initiation without causing cytotoxicity. We suggest that blockade HIF-1a and reforming energy metabolism with auraptene is an effective approach for suspension RCC progression.

## INTRODUCTION

Nephrectomy is the first-line strategy for curative treatment in the early stage of renal cell carcinoma (RCC). Yet despite this surgery, 30% of RCC patients face the risk of metastasis to distant sites, including liver, lymph node, brain, bone and pancreas, resulting in a 5-year decrease in survival [1–3]. The aggressiveness of these cancers is accompanied by a metabolic switch toward aerobic glycolysis, called the Warburg effect, which reduces reactive oxygen species (ROS) levels and increases the supply of ATP [4–6]. As such, this switch is considered indispensable for the migration of RCC cells. Some types of solid cancer with a highly metastatic phenotype, such as RCC and breast cancer, require a high intracellular antioxidant capacity and enhanced ATP generation to support tumor cell migration [7]. Although, it has been reported that mitochondrial complex I inhibitors such as rollinistatin and bullatacin, decrease mitochondrial respiration and ATP generation, thereby inhibiting metastasis of lung and breast cancer cells [8], their effect on RCC has not been studied. Accordingly, suppression of ATP generation by targeting both glycolysis and mitochondrial respiration is seen as essential for blocking cancer progression [9]. However, efforts to reverse glycolytic changes in cancers and inhibit mitochondrial respiration are known to produce cytotoxic effects [9, 10]. Moreover, no modulators with dual mitochondria- and glycolysis-targeting activity that are effective against RCC have been reported.

Auraptene, a natural compound initially isolated and purified from citrus fruit, is known as a mitochondrial complex I inhibitor [11]. In rodent models, auraptene has shown inhibitory effects on mammary and oral carcinogenesis and metastasis of melanoma through modulation of metabolism-related processes, such as hypoxic signaling [12–14]. Auraptene has also been reported to possess apoptogenic activity toward human acute leukemia cells [15]. Although auraptene is among the most useful drugs for regulating tumor progression through modulation of energy metabolism, its efficacy against RCC has not been tested.

Hereditary forms of RCC, including von Hippel–Lindau (VHL) syndrome, account for 2–4% of total RCC occurrence [16]. The tumor suppressor VHL is an E3 ubiquitin ligase responsible for regulating the degradation of hypoxia-inducible factor 1α (HIF-1α). In patients with congenital VHL, a mutation in the *VHL* gene that inactivates VHL leads to dysregulation of HIF-1α, setting the stage for the development of RCC [17, 18]. Loss or mutation of the remaining wild-type allele results in constitutive expression of HIF-1α, which is a key modulator of tumor metabolism. Induction of HIF-1α by hypoxia—a characteristic feature of the tumor environment—promotes the transcription of target genes that lead to invasiveness, metabolic shift, angiogenesis, and metastatic potential [19–21]. Overexpression of HIF-1α is correlated with metastasis of hepatocellular carcinoma cells [22]. In the current study, we selected auraptene as a candidate modulator of energy metabolism in RCC4 cells through direct targeting of HIF-1α and mitochondrial respiration, examining its suppressive effects on cancer progression.

## RESULTS

### Auraptene inhibits glycolytic and mitochondrial metabolism, but does not affect cell growth

**Figure 1:**
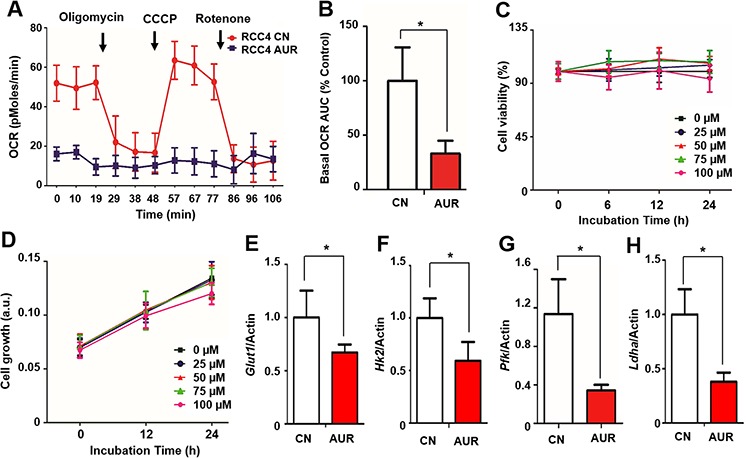
Auraptene significantly reduces the OCR of RCC cells and disrupts transcription of HIF-1α target genes without affecting cell viability **A.** The OCR of RCC4 cells was measured using an XF24 analyzer after incubation with DMSO or 100 μM auraptene for 24 h. Arrows indicate times at which mitochondrial inhibitors (2 μg/ml oligomycin, 5 μM CCCP, and 2 μM rotenone) were added to media. **B.** Basal OCR area under the curve was calculated using XF24 software. **C, D.** Effects of 0, 25, 50, 75, and 100 μM auraptene on RCC4 cell viability after the indicated times was determined by MTT assay and SRB, respectively. Viability is presented as a percentage relative to zero time. **E–H.** mRNA expression of *GLUT, HK2, LDHA* and *PFK* in RCC4 cells cultured in the presence or absence of 100 μM auraptene for 24 h was assessed by qPCR. Values are expressed as fold change. β-Actin was used as control. Data are presented as means ± SD (bars) of triplicate samples (*P < 0.05).

Because auraptene is an inhibitor of mitochondrial complex I [11], it may be a candidate for the regulation of energy metabolism in RCC. To assess whether mitochondrial oxidative phosphorylation (OXPHOS) in RCC is affected by auraptene, RCC4 cells were incubated with 100 μM auraptene or DMSO (vehicle control) for 24 h and the effects on basal OCR were determined using an XF-24 analyzer. Auraptene significantly inhibited mitochondrial respiration (Fig. 1A), decreasing the basal OCR area under the curve by about 67% compared to that in DMSO-treated RCC4 cells (Fig. 1B) but did not change in basal ECAR level (Fig. S1). Despite strongly inhibiting mitochondrial complex I, auraptene had no effect on cell viability, as determined by MTT assay (Fig. 1C). Similarly, auraptene treatment did not affect cell growth in the sulforhodamine B (SRB) assay (Fig. 1D).

As auraptene inhibits mitochondrial respiration, cells would require a supply of glycolytic ATP to maintain growth. We found that the mRNAs encoding glucose transporter 1 (GLUT1), hexokinase 2 (HK2), phosphofructokinase (PFK) and lactate dehydrogenase A (LDHA), all key enzymes in the glycolytic pathway, were expressed in RCC4 cells, but quantitative RT-PCR (qPCR) showed that their expression was reduced 30–60% in the presence of auraptene (*p* < 0.05; Fig. 1E–1H). Nevertheless, intracellular ATP content and ADP/ATP ratio were unchanged by auraptene treatment (Fig. S2). Collectively, these results indicate that auraptene significantly reduces mitochondrial respiration in RCC cells and slightly suppresses the transcription of glycolytic pathway-related genes without affecting cell growth.

### Auraptene decreases RCC4 cell motility and inhibits tube formation by HUVECs

**Figure 2:**
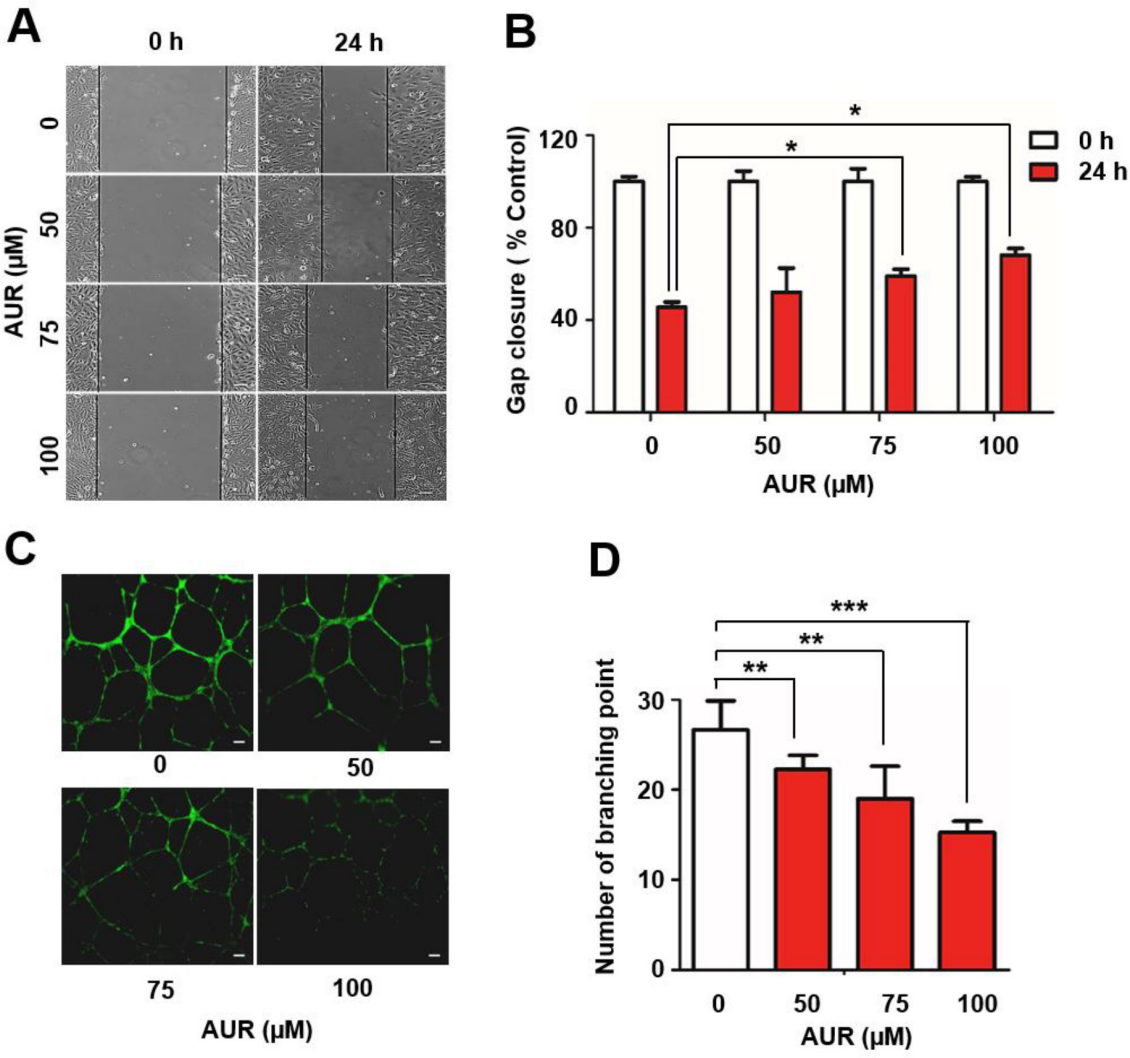
Auraptene delays RCC4 cell migration and inhibits tube formation by HUVECs **A.** RCC4 cell wound-healing assays were performed in the presence or absence of 100 μM auraptene after 24 h. **B.** Gap closure was measured and presented as gap distance relative to controls (as a percentage). **C.** Effects of auraptene on angiogenesis were verified by HUVEC tube-formation assays. HUVECs were plated on Matrigel and incubated under hypoxic (1% O_2_) conditions. Tube formation was visualized with the fluorescent dye, calcein-AM, 24 h after auraptene treatment. Scale bars: 10 μm. **D.** The number of branching points was counted. Data are presented as means ± SD (bars) of triplicate samples (*P < 0.05, **P < 0.01; ***P < 0.001).

A reduction in energy metabolism can affect cell motility and angiogenesis as well as cell proliferation [23]. To test whether auraptene has an inhibitory effect on RCC4 cell motility, we performed wound-healing assays. RCC4 cells were cultured in the presence of different concentrations of auraptene or DMSO; then, cell monolayers were wounded by scoring with a pipet tip and the gap width was measured after 24 h of treatment. Gap closure was decreased by about 20% in auraptene treated cells compared with DMSO-treated cells (Fig. 2A, 2B), suggesting that auraptene effectively disrupted tumor cell migration. Tube formation by HUVECs is an indicator of tumor angiogenesis [20, 24]. We assessed the effect of different concentrations of auraptene (0, 50, 75 and 100 μM) on tube formation by HUVECs. HUVECs cultured on Matrigel under hypoxic conditions formed a capillary tube network. Auraptene inhibited tube formation by Matrigel-cultured HUVECs compared with DMSO-treated cells, decreasing the number of branch points at the lowest concentration tested (50 μM) and further decreasing the number of branch points at 75 μM (by ~7) and 100 μM (by ~10) (Fig. 2C, 2D). These results indicate that auraptene significantly reduces motility of RCC4 cells and effectively inhibits tube formation by HUVECs.

### Auraptene inhibits VEGF-induced neovascularization *in vivo*

**Figure 3:**
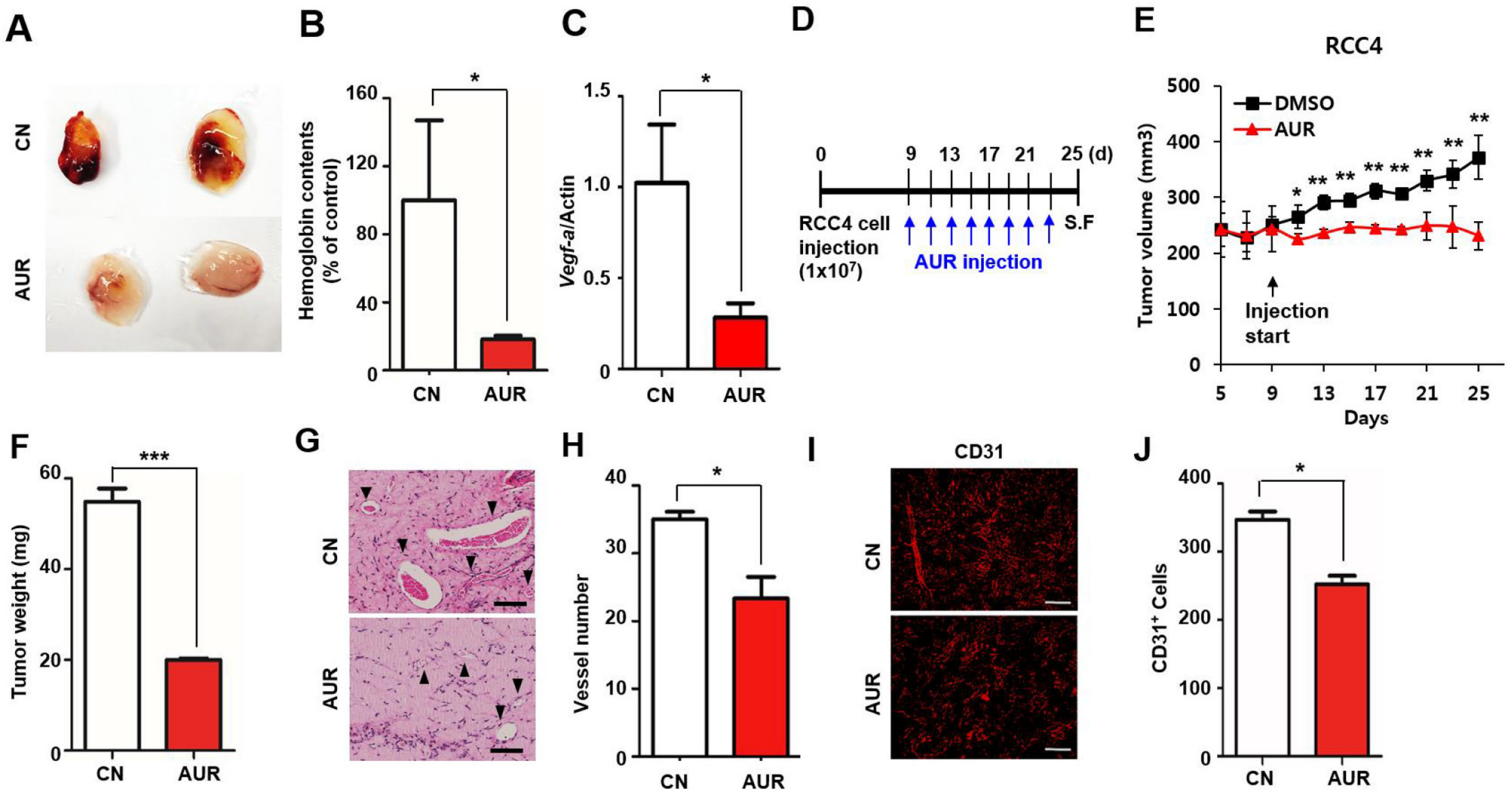
Auraptene inhibits VEGF-induced angiogenesis *in vivo* **A.** VEGF-induced angiogenesis was assessed by the Matrigel plug assay. A Matrigel mixture containing DMSO or 100 μM auraptene was subcutaneously injected into 6 week old male immunodeficient mice (*n* = 5 per group). After 7 days, the Matrigel plug was detached from the skin and photographed. **B.** Blood extracted from Matrigel was added to Drabkin's solution, and the absorbance at OD 450 nm was measured. The hemoglobin contents of each plug were represented as % of control. **C.** *VEGF-A* mRNA expression in RCC4 cells cultured in the presence or absence of 100 μM auraptene for 24 h, as assessed by qPCR analysis. Values were normalized to those of β-actin. Data are presented as means ± SDs (bars) of triplicate samples (*P < 0.05). **D.** Effect of auraptene on RCC4 xenografts. RCC4 cells (1×10^7^ RCC4) were subcutaneously injected to the flank of 5 week old nude mice. After 9 days, auraptene or DMSO was injected into the tumor every other day (*n* = 10 per group). **E.** tumor size measured by calipers every other day and calculation of tumor volumes. **F.** tumor weight. **G.** H&E staining of vessels from xenografts after 25 days. Arrows indicate the vessels. **H.** numbers of vessels. **I.** immunofluorescence images of CD31 of DMSO- and auraptene-injected tumors. **J.** Counting of CD31 positive cells. The graph represents the mean ± SEM (bars) of 5 mice in each group. Scale bar in G is 100 μm and H is 200 μm.

To verify that auraptene has anti-angiogenic activity, we performed the Matrigel plug assay *in vivo*. A mixture of Matrigel, vascular endothelial growth factor (VEGF) and either DMSO or 100 μM auraptene was subcutaneously injected to the flanks of nude mice. Seven days later, the plugs was detached from the skin and visualized. In the absence of auraptene, the plugs had a red color due to the infiltration of vessels (Fig. 3A). Neovascularization was quantified by measuring the hemoglobin content of the plugs, with the hemoglobin content of each plug represented as a percentage of control. Plugs in the auraptene treated group had a 5-fold lower hemoglobin content than control plugs (Fig. 3B), and a lower density of infiltrated blood vessels. VEGF-induced angiogenesis is crucial for tumor progression [24, 25] and antiangiogenic agents have been used to inhibit aggressive tumor spread. We verified whether auraptene affects the transcription of *Vegf-a*, finding that auraptene reduced *Vegf-a* mRNA level by ~70%, as shown by qPCR analysis (Fig. 3C). These results indicate that auraptene effectively inhibited VEGF-induced angiogenesis *in vivo*.

To determine whether auraptene inhibited angiogenesis *in vivo*, 1×10^7^ RCC4 cells were subcutaneously injected into 5 week old nude mice, followed nine days later by the intratumoral injection of auraptene every other day (Fig. 3D). We found that auraptene treated mice had reduced tumor growth, whereas tumors injected with DMSO showed a gradual increase in size (Fig. 3E). After 25 days, the mice were sacrificed and tumor weight and vascularization status were analyzed. As expected, tumor weight (Fig. 3F) and the number of well-vascularized vessels (Fig. 3G, 3H) were each about one-third lower in auraptene- than in DMSO-treated mice. Immature vessels with reduced diameter, blood leakage represented by red blood cells and stained with brown colored hemosiderin were observed in auraptene injected group compared to the well-formed vessels of DMSO-treated mice with basement membrane and pericytes surrounding vessel wall. (Fig. 3G). Consistent with H&E staining results, immunofluorescence showed that auraptene reduced the number of CD31 positive cells but increased heterogeneity (Fig. 3I, 3J). Taken together, these findings indicate that auraptene can inhibit vessel formation, suppressing tumor cell growth.

### Auraptene induces HIF-1α degradation in a VHL-independent manner, and HIF-1α knockdown delays RCC4 cell migration

**Figure 4:**
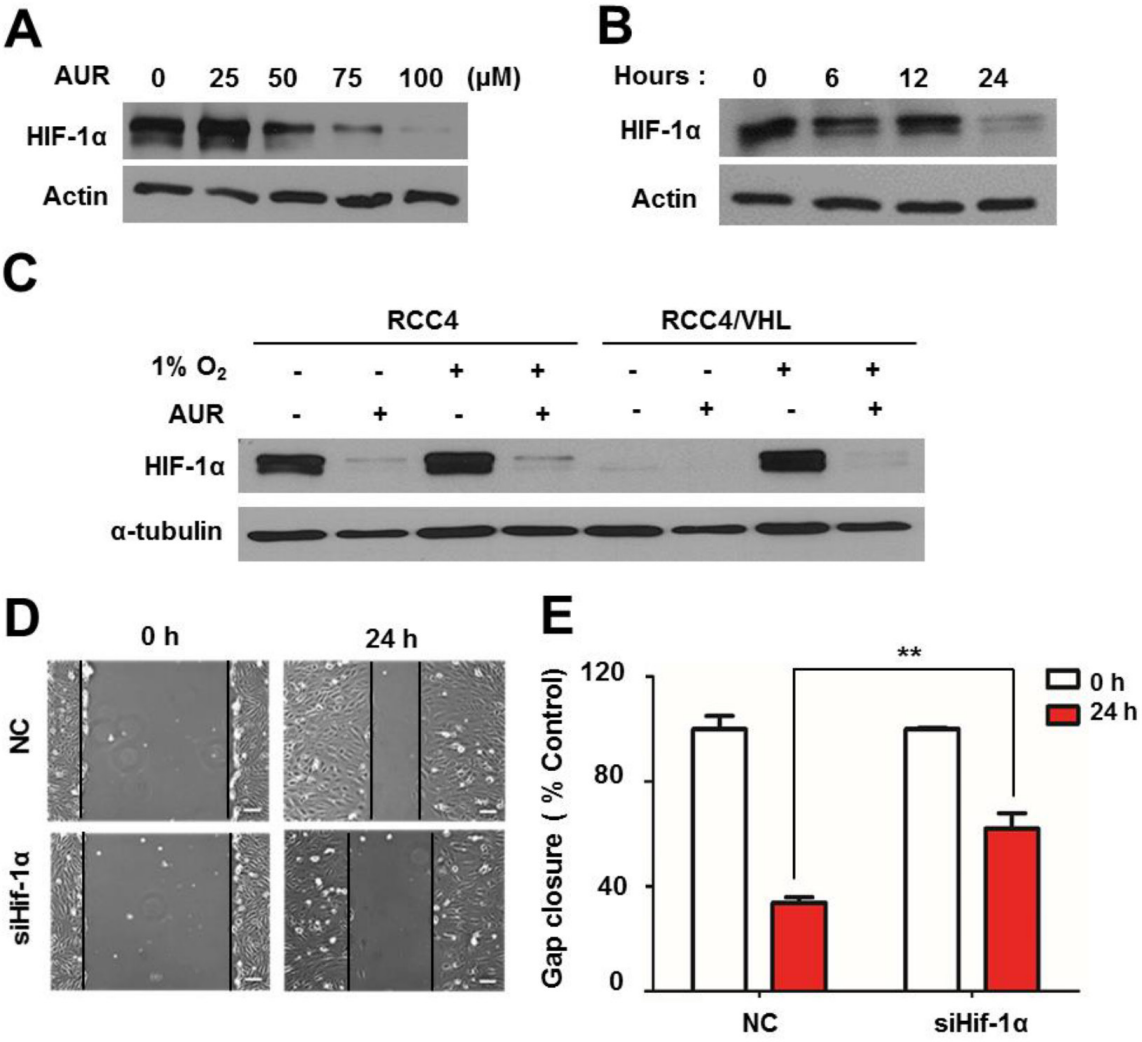
Auraptene induces HIF-1α degradation in a VHL-independent manner, and siRNA-mediated HIF-1α knockdown delays RCC4 cell migration **A.** RCC4 cells were incubated for 24 h in the absence or presence of 0, 25, 50, 75 or 100 μM auraptene (AUR). **B.** RCC4 cells were cultured in the absence or presence of AUR (100 μM) for 0, 6, 12 or 24 h. **C.** RCC4 cells and RCC4/VHL cells were incubated for 24 h in the presence or absence of 100 μM auraptene under normoxic or hypoxic conditions. Protein levels of HIF-1α and α-tubulin were assessed by Western blotting. **D.** Effects of siHif-1α on cell migration were verified. **E.** Average distance to the opposing side of the wound was measured 24 h after siHif-1α transfection and expressed as gap distance relative to controls (as a percentage). Data are presented as means ± SD (bars) of triplicate samples (**P < 0.01). NC, negative control.

HIF-1α is involved in promoting vessel formation through induction of VEGF expression and enhancing tumor growth through upregulation of glycolytic pathway-related genes [20, 26, 27]. To test the role of HIF-1α in mediating the effects of aurapene, we first assessed HIF-1α transcription levels by qPCR, but found no significant change in HIF-1α mRNA level with auraptene treatment (Fig. S3). Next, we assessed whether auraptene affected HIF-1α protein levels in RCC4 cells, which constitutively express HIF-1α protein owing to a deficiency of VHL [17, 28]. Unlike the case for murine embryonic fibroblasts (MEFs), HepG2 human hepatic carcinoma cells and MCF-7 human breast cancer, HIF-1α was notably elevated in RCC4 cells, even under normoxic conditions, and was prominently localized in the nucleus (Fig. S4A, S4B). In RCC4 cells treated for 24 h, auraptene decreased HIF-1α protein levels in a concentration-dependent manner (Fig. 4A). Over this same time frame, treatment with 100 μM auraptene resulted in the time-dependent degradation of HIF-1α (Fig. 4B). To test whether auraptene affected HIF-1α induction in the context of hypoxic conditions representative of the tumor environment, we reconstituted VHL in RCC4 cells, which lack endogenous VHL, and assessed the effects of auraptene (100 μM) on these RCC4/VHL cells under normoxic (20% O_2_) and hypoxic (1% O_2_) conditions. As shown in Fig. 4C, auraptene strongly decreased HIF-1α protein levels in RCC4/VHL cells under hypoxic conditions, and also inhibited mitochondrial respiration (Fig. S5). These results suggest that auraptene induces HIF-1α down-regulation regardless of the presence or absence of VHL. To determine whether the inhibitory effect of auraptene on cell migration is a consequence of HIF-1α inhibition, we transfected RCC4 cells with a siRNA targeting a Hif-1α consensus RNA and performed wound-healing assays. Knockdown of HIF-1α was confirmed by Western blotting (Fig. S6). Following wounding and treatment with auraptene, gap distance was increased more than 2-folds in siHif-1α–transfected cells compared with cells transfected with a scrambled control siRNA (Fig. 4D, 4E).

### Auraptene promotes HIF-1α protein degradation by inhibition of translation initiation

**Figure 5:**
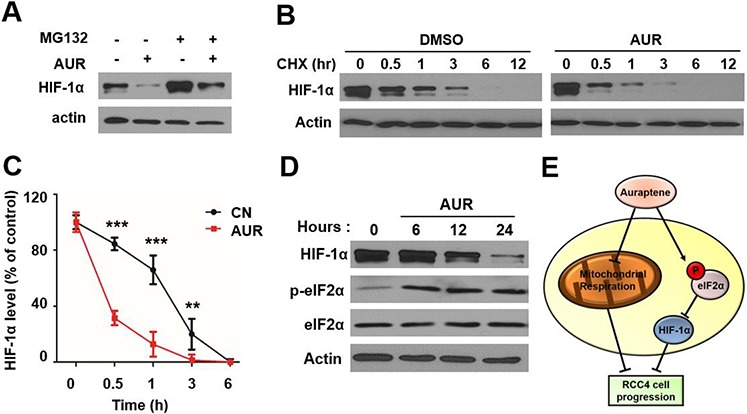
Auraptene significantly decreases HIF-1α protein through translational inhibition **A.** RCC4 cells were incubated in the absence or presence of 100 μM auraptene and 20 μM MG132 for 24 h. **B.** RCC4 cells were cultured in the absence or presence of 100 μM auraptene and 40 μg/mL CHX, added to media at the indicated incubation times. HIF-1α and β-actin levels were analyzed by Western blotting. **C.** Band intensity was measured using the ImageJ program. Data are presented as means ± SD (bars) of triplicate samples (**P < 0.01; ***P < 0.001). **D.** Protein levels of HIF-1α, total eIF2α, and p-eIF2α were determined by Western blotting after administration of auraptene. β-Actin was used as a loading control. **E.** Schematic depiction of inhibition of angiogenesis and motility of RCC cells by auraptene through regulation of HIF-1α and eIF2α.

To determine whether the decrease in HIF-1α levels was attributable an increase in HIF-1α protein degradation (decreased half-life) or a decrease in translation, we first evaluated proteasome-mediated degradation of HIF-1α. In RCC4 cells treated with DMSO (vehicle control), co-treatment the proteasome inhibitor MG132 resulted in the accumulation of HIF-1α. Notably, however, even with the proteasome inhibited with MG132, co-treatment with auraptene still significantly decreased HIF-1α levels compared with DMSO co-treatment (Fig. 5A). Interactions with heat shock protein 90 (HSP90) are important for HIF-1α protein stability, with dissociation from HSP90 leading to proteasome-mediated degradation of HIF-1α [29, 30]. To determine whether auraptene acted by interfering with the association of HIF-1α with HSP90, we overexpressed HSP90 in RCC4 cells by transfecting cells with a FLAG-HSP90 expression construct and tested the interaction of HIF-1α with HSP90 in the presence and absence of auraptene. We found that auraptene had no effect on the association of HIF-1α with HSP90 (Fig. S7). Taken together, these data suggest that auraptene-induced HIF-1α down-regulation is not dependent on proteasome-mediated degradation. We did find, however, that auraptene decreased the half-life of HIF-1α protein in RCC cells, showing that, in the presence of cycloheximide to inhibit de novo protein synthesis, HIF-1α protein levels decreased more rapidly in the presence of auraptene (Fig. 5B, 5C). Finally, we examined whether the effects of auraptene on HIF-1α levels involved eukaryotic translation initiation factor 2α (eIF2α), a component of the eIF2 ternary complex that is known to initiate HIF-1α gene translation [31]. In RCC4 cells and HeLa cells grown under hypoxic conditions, treatment with auraptene significantly increased the levels of phosphorylated eIF2α (p-eIF2α) (Fig. 5D and Fig. S8). These results suggest that auraptene down-regulates HIF-1α protein by inhibiting its translation initiation rather than by promoting its degradation (Fig. 5E). Notably, auraptene did not affect cleavage of poly-(ADP-ribose) polymerase (PARP), an apoptosis marker protein, in HeLa cells (Fig. S8), further supporting the conclusion that auraptene is not cytotoxic.

## DISCUSSION

About 30% of patients with RCC, a potentially fatal type of solid tumor, suffer from uncontrolled metastasis and chemoresistance [1–3]. RCC metastasis is starting from enhanced migration and it is not restricted to lymph nodes around the kidney, but can spread to distant organs, such as the pancreas, bone, and brain. To date, interferon (IFN), sunitinib and bevacizumab, and interleukin (IL)-2 have been the main options for treating RCC and preventing recurrence. Unfortunately, response rates for these drugs in preventing metastasis and recurrence are only ~15% [32]. Given this limited efficacy, clinicians have turned to other therapeutics, including sorafenib and pazopanib—inhibitors of vascular endothelial growth factor A (VEGF-A) that target tumor angiogenesis—and multi-tyrosine kinase inhibitors that target platelet-derived growth factor (PDGFR)-α or VEGFR-1. However, these drugs are unable to overcome chemoresistance and leukopenia owing to their cytotoxic effects [33–35]. In the present study, we investigated the anticancer properties of auraptene, isolated from citrus fruit, against RCC. We found that auraptene significantly suppressed tumor energy metabolism, leading to inhibition of motility and it did so without causing cytotoxicity.

Cancer cells favor anaerobic glycolysis as the metabolic pathway for obtaining ATP, although mitochondrial OXPHOS still provides low amounts of ATP [9]. This preference has motivated trials to reverse the aberrant metabolism in cancer cells using drugs such as the α-tocopherol derivative, ESeroS-GS [9, 36]. However, it has been reported that α-tocopherol derivatives and combined administration of glycolysis and mitochondrial inhibitors are associated with cytotoxicity [8, 37]. In contrast, one previous study reported that administration of auraptene was cytotoxic toward breast cancer cells only under serum-starved conditions. Furthermore, against the Jurkat T cell line, auraptene showed cytotoxic effects only at high concentrations (>150 μM) [15]. According to the previous report, auraptene content of dried peel powder of *Citrus Kawachiensis* was defined to 4.07 ± 0.033 mg/g [38]. They daily administered 32 and 64 mg/kg of auraptene to mouse orally and it showed beneficial effect for defending inflammation. We have mostly used 100 μM auraptene in current study, which is obtainable using the concentrated powder and it did not show toxicity at this concentration. Notably, although auraptene treatment suppressed mitochondrial respiration and glycolytic pathway-related genes in RCC cells, it did not affect cell viability at concentrations less than 100 μM. In line with the cell viability result, ATP content and ADP/ATP ratio did not changed and we can predict the alternative pathway of glycolysis compensate energy depletion such as TCA cycle and beta oxidation (Fig. S9). Moreover we cannot find any Tunnel positive cells by treatment of auraptene *in vivo* (Fig. S10A, S10B). Accordingly, auraptene could be a candidate for inhibiting cancer progression with no associated cytotoxicity.

The enhancement of VEGF-induced angiogenesis is observed in stages of tumor progression and therapeutic strategies targeting neovascularization of tumor has been tried [25]. So, we assessed whether auraptene had an antiangiogenic effect. We found that auraptene directly decrease VEGF mRNA expression. Importantly, auraptene effectively inhibited tube formation by HUVEC cells under hypoxic condition. Also, auraptene inhibited VEGF-induced neovascularization by 10-fold *in vivo.* Tumor cell motility and neovascularization are necessary for RCC progression, a process that requires rapid availability of an abundant energy supply. According to the Warburg effect, most of the energy used is supplied by glycolysis. Consistent with this hypothesis, HK2 and GLUT1 are also highly expressed in many tumors, including RCCs, and mediate enzymatic and transport processes involved in glycolysis [39]. Furthermore, LDHA contributes to aerobic glycolysis in tumors. The abovementioned glycolysis-related genes are targeted by the transcription factor, HIF-1α. Unlike other tumors, which express HIF-1α only under hypoxic conditions, RCCs express high levels of HIF-1α under normoxic conditions. We found that auraptene abolished HIF-1α protein translation and reduced transcription of HIF-1α target genes, including *GLUT2, HK2* and *LDHA,* which are involved in regulating tumor metabolism [5, 19, 21]. This ablation of HIF-1α by auraptene was further associated with inhibition of angiogenesis, as reflected in reduced endothelial tube formation and decreased expression of *VEGF*, as well as suppression of RCC cell migration. As expected, siRNA-mediated HIF-1α knockdown resulted in inhibition of RCC cell motility. Consistent with these findings, small molecule inhibitors of HIF-1α such as PX-478 and geldanamycin have been shown to reduce tumor cell proliferation and angiogenesis [29, 40, 41]. However, unlike auraptene, these compounds also induce apoptosis.

EIF2α is known to regulate HIF-1α gene translation [31]. Phosphorylation on Ser51 of the eIF2α subunit blocks formation of the eIF2 ternary complex, resulting in inhibition of HIF-1α translation [40]. Aminoflavone, a ligand of the aryl hydrocarbon receptor, is known to repress HIF-1α translation in human breast cancer cells by inducing eIF2α phosphorylation [42]. Hydrogen sulfide (H_2_S) is also reported to regulate HIF-1α through eIF2α phosphorylation. This study showed that auraptene significantly induced phosphorylation of eIF2α beginning at 6 h, and that acute treatment with auraptene rapidly (within 7 min) inhibited mitochondrial respiration (Fig. S11A, S11B) and simultaneously increased ECAR (Fig. S11C, S11D). Although we did not specifically address the temporal relationship between mitochondrial inhibitory function and eIF2α phosphorylation, we expect that suppression of mitochondrial energy metabolism proceeds to HIF-1α degradation within 6 h. Inhibitors of HIF-1α transactivation carry a risk of causing coagulative necrosis and cytotoxicity [43]. However, because HIF-1α is not the initial target, auraptene can more effectively inhibit cellular motility and tube formation without causing cytotoxicity. Auraptene effect on HIF-1α down-regulation was also verified using HeLa cells grown under hypoxic (1% O_2_) conditions. Additionally, auraptene significantly induce phosphorylation of eIF2α in a dose dependent manner (Fig. S8). These finding suggests that anticancer property of auraptene through inhibition of HIF-1α is not restricted to renal cell carcinoma. Taken together, our results demonstrate that auraptene exerts a suppressive effect on the progression of RCC4 cells by strongly inhibiting mitochondrial respiration and directly targeting HIF-1α signaling, without producing cytotoxic effects.

## MATERIALS AND METHODS

### Cell culture

RCC4 and RCC4/VHL human RCC cells were purchased from ECACC (Salisbury, UK). As instructors indicated, RCC4 cells were stably transfected with pcDNA3 vector and RCC4/VHL cells were transfected with pcDNA3-VHL, conferring neomycin resistance. Cells were maintained in Dulbecco's Modified Eagle Medium (DMEM) containing 10% fetal bovine serum (FBS), 1% penicillin/streptomycin, and 100 μg/ml G418 at 37°C in a humidified 5% CO_2_/21% O_2_ environment. Low-oxygen conditions were created by adjusting the environment to 1% oxygen, 5% CO_2_, and 94% N_2_ using a hypoxia chamber (New Brunswick Scientific, CT, USA). Auraptene was dissolved in dimethyl sulfoxide (DMSO). Cycloheximide was purchased from Sigma-Aldrich, and MG132 was from Calbiochem. Knockdown of HIF-1α in RCC4 was achieved by transfecting cells for 24 h with small interfering RNA (siRNA) using G-fectin (Genolution Pharmaceuticals, Seoul, Republic of Korea), as described by the manufacturer.

### Determination of oxygen consumption rate

Oxygen consumption rate (OCR) was analyzed using an XF24 analyzer (Seahorse, MA, USA). RCC4 or RCC4/VHL cells were plated at 5 × 10^3^ cells/well and incubated with DMSO or 100 μM auraptene for 24 h. After measuring basal OCR, the mitochondrial ATP synthase inhibitor, oligomycin (2 μg/ml), was added, followed sequentially by addition of the mitochondrial uncoupler, carbonyl cyanide 3-chlorophenylhydrazone (CCCP; 5 μM), and the mitochondrial complex I inhibitor, rotenone (2 μM). OCR was measured after addition of each agent.

### Measurement of cell viability

RCC4 cells were seeded in 96-well plates and incubated in media containing different concentrations of auraptene (0, 25, 50, 75 and 100 μM) for 6, 12 and 24 h at 37°C under normoxic (5% CO_2_/21% O_2_) conditions. The media were removed and serum-free medium containing 0.5 mg/ml 3-(4, 5-dimethylthiazol-2-yl)-2, 5-diphenyltetrazolium bromide (MTT) was added. After 1 h, the MTT-containing media were removed and DMSO was added to dissolve formazan crystals. Absorbance was measured at 570 nm using a Multiskan Ascent plate reader (Thermo Scientific, MA, USA). In the sulforhodamine B assay, RCC4 cells (4×10^3^ cells per well) were seeded in triplicate in 96-well plates and incubated overnight. To each well as added medium containing 0, 25, 50, 75 and 100 μM auraptene, followed by incubation for 12 or 24 h. The media were discarded and cells were fixed with 10% TSA at 4°C for 1 h. After washing with distilled water, the cells were incubated with 0.4% SRB (Sigma-Aldrich, SG, Switzerland) solution at room temperature for 20 min. The plates were washed with 1% acetic acid five times and dried in air. Proteins were resolved with 10 mM unbuffered Tris and absorbance was read at 490 nm using a Multiskan Ascent plate reader.

### RNA isolation and real-time PCR

Total RNA was isolated from cells using isol-RNA lysis reagent (5 PRIME, CA, USA). cDNA way synthesized from total RNA by reverse transcription using an M-MLV reverse transcriptase system (Invitrogen, CA, USA), according to the manufacturer's instructions. Real-time polymerase chain reaction (PCR) was performed using a SYBR mix and a Rotor-Gene 6000 Real-Time Rotary Analyzer system (Corbett Life Science, Venlo, Netherlands). The following primer pairs were used: *HIF-1α*, 5′-CCA CCT ATG ACC TGC TTG GT-3′ (forward) and 5′-TAT CCA GGC TGT GTC GAC TG-3′ (reverse); *LDHA*, 5′-TGT GCC TGT ATG GAG TGG AA-3′ (forward) and 5′-AGC ACT CTC AAC CAC CTG CT-3′ (reverse); *GLUT1*, 5′-GCC CTG GAT GTC CTA TCT GA-3′ (forward) and 5′-CCC ACG ATG AAG TTT GAG GT-3′ (reverse); *HK2,* 5′-TAG GGC TTG AGA GCA CCT GT-3′ (forward) and 5′-CCA CAC CCA CTG TCA CTT TG-3′ (reverse); *VEGF,* 5′-CCT TGC TGC TCT ACC TCC AC-3′ (forward) and 5′-CAC ACA GGA TGG CTT GAA GA-3′ (reverse); and *β-actin*, 5′-GTC GTA CCA CTG GCA TTG TG-3′ (forward) and 5′-CTC TCA GCT GTG GTG GTG AA-3′ (reverse); *PFK*, 5′ -GAA GAG CCC TTC GAC ATC AG-3′ (forward) and 5′–TCT TCC TGC AGT CAA ACA CG -3′ (forward).

### Wound-healing assay

RCC4 cells were cultured in the presence or absence of auraptene, then, monolayers were scratched with a pipet tip. Gap width was measured after 24 h using ImageJ program.

### Endothelial cell tube-formation assay

Human umbilical vein endothelial cells (HUVECs) in EBM-2 media supplemented with 0.1% FBS were plated on Matrigel basement membranes (BD Biosciences, NJ, USA) and incubated for 8 h under hypoxic conditions. Auraptene was added to media, after which cells were stained with calcein-AM (Sigma-Aldrich, SG, Switzerland). Intracellular calcein-AM fluorescence (n = 10 slides/condition) was imaged using an IX70 fluorescence microscope (Olympus, Tokyo, Japan).

### Matrigel plug assay

Matrigel (BD Bioscience, MA, USA) was thawed on 4°C, then, 400 μl of Matrigel was mixed with 200 μl serum-free DMEM media, 50 ng VEGF (R&D Systems, MN, USA) and 40 u heparin (Huons, Seongnam, Republic of Korea). DMSO or 100 uM auraptene was added to the mixture. Then, Matrigel mixture was subcutaneously injected to the flank of 6 weeks old male immune-deficient mice (*n* = 5, each group). Mouse were purchased from Japan SLC (SLC, Hamamatsu, Japan) and kept in pathogen free facility. Animal experiment was approved by the Institutional Animal Care and Use Committee of Chungnam National University. The ethical approval number is CNU-00356. Neovascularization was quantified by measuring hemoglobin content using Drabkin's reagents (Sigma-Aldrich, SG, Switzerland) after 7 days of injection. After removing plug from skin, plug was homogenized at 4°C for 18 hours and centrifuged at 10,000 rpm at 4°C. 5 ml of Drabkin's solution was added to the supernatant and incubated at RT for 15 minutes. The absorbance at 540 nm was measured in ELISA plate reader (Molecular Devices, CA, USA).

### Xenograft model

To generate RCC4 xenografts, 1×10^7^ cells in 100 μM serum free DMEM were mixed with an equal volume of Matrigel (BD Biosciences, NJ, USA), and the mixture was injected subcutaneously into 5 week old male nude mice (*n* = 10 per group). Beginning 9 days later, auraptene or DMSO diluted in 50 μl saline was intratumorally injected every other day. Tumor size was measured with calipers and tumor volume was calculated using the formula: width^2^x length/2. The mice were sacrificed and the tumor tissue was fixed in 4% paraformaldehyde for immunohistochemistry.

### Immunohistochemistry

Tumors of DMSO- or auraptene- treated mice was fixed with 4% paraformaldehyde and paraffin embedded. Sections were prepared for hematoxylin and eosin (H&E) staining. After deparaffinization, sections were stained with Harris hematoxylin for 4 minutes. Then, sections were washed with running water 4 min, stained with eosin for 2 min and serial washing with ethanol and xylene was proceeded. For CD31 immunofluorescence staining, sections were deparaffinized and blocked for 30 min with protein blocking buffer (DAKO, Glostrup, Denmark). After incubation with CD31 antibody (Abcam, MA, USA), sections were washed with TBST buffer and incubated with secondary fluorescence antibody. Intracellular CD31 fluorescence (*n* = 10 slides/ condition) was visualized using an IX70 fluorescence microscope (Olympus, Tokyo, Japan).

### Western blotting

Cells were lysed with RIPA buffer (50 mM Tris–HCl pH 7.5, 150 mM NaCl, 1% Nonidet P-40, 0.5% deoxycholate, 0.1% SDS) containing protease inhibitor cocktail (Roche, Basel, Switzerland), with or without phosphatase inhibitor (Roche). Equal amounts of protein were resolved by sodium dodecyl sulfate-polyacrylamide gel electrophoresis (SDS-PAGE) and then transferred to nitrocellulose membranes (Pall Corporation, NY, USA). Membranes were probed first with anti-HIF-1α (Novus Biologicals, CO, USA), anti-rabbit β-actin (Santa Cruz Biotechnology, CA, USA), anti-α-tubulin (Sigma-Aldrich, SG, Switzerland), anti-rabbit eIF2α (MyBioSource, CA, USA), and anti-rabbit phospho-eIF2α (Merck Millipore, MA, USA) primary antibodies, and then with horseradish peroxidase (HRP)-conjugated anti-mouse IgG (Pierce Biotechnology, MA, USA) or anti-rabbit IgG (Calbiochem, MA, USA) secondary antibody, as appropriate. Immunoreactive proteins were detected using an enhanced chemiluminescence (ECL) system (Amersham Biosciences, Buckinghamshire, England).

### Statistical analysis

Results are presented as means ± standard deviation (SD) of triplicate determinations. The statistical significance of differences between two means was determined by one-tailed Student's *t*-test. A *P*-value < 0.05 was considered significant; *P*-values for specific comparisons are indicated in figure legends.

## SUPPLEMENTARY FIGURES


