# Supplementary material for: Suppression of mitochondrial respiration with auraptene inhibits the progression of renal cell carcinoma: involvement of HIF-1α degradation
Source: Oncotarget. 2015 Oct 12;6(35):38127–38. doi: 10.18632/oncotarget.5511 (PMC4741988; doi:10.18632/oncotarget.5511)
Supplement: Supplementary file 1 [file oncotarget-06-38127-s001.pdf]

SUPPLEMENTARY DATA

ADP/ATP assay

ADP/ATP ratios were measured using appropriate assay kits (Abcam, Cambridge, UK). Briefly, RCC4 cells were cultured in triplicate for 24 h, followed by the addition of DMSO or auraptene for 24 h. The media were discarded, and nucleotide releasing buffer was added to each well. After incubation at room temperature for 5 min, ATP monitoring enzyme was added to each well and luminescence was measured with a luminometer (Berthold Technologies, Bad Wildbad, Germany). To each well was added 1X ADP converting enzyme, ADP luminescence was measured, and ADP/ATP ratio was calculated.

Immunofluorescence staining

RCC4 cells were grown on cover slips treated with ethanol and nitric acid solution. After treating with auraptene, cells were fixed with 4% paraformaldehyde for 15 min at room temperature (RT), then permeabilized by incubating with 0.25% Triton X-100 containing phosphate buffered saline (PBS) for 10 min. Fixed, permeabilized cells were incubated with anti-mouse HIF-1α antibody (BD Transduction, NJ, USA) in PBS at 4°C for 16 h. Cells were washed with PBS, then incubated for 1 h at RT with anti-mouse Alexa Fluor 488-conjugated secondary antibody diluted in PBS. Cells were incubated for 5 min at RT with 4′, 6-diamidino-2-phenylindole (DAPI; Life Technologies, CA, USA) to stain nuclei, then mounted on a slide. HIF-1α and DAPI were observed using an LSM 510 META microscope (Carl Zeiss AG, Jena, Germany).

Tunnel staining

Tumor slice was prepared for Tunnel assay. After deparaffinization, Tunnel staining (*n* = 10 slides/condition) was performed with Tunnel assay kit (Promega, WI, USA) according to manufacturer's instruction. Tunnel immunofluorescence was visualized using an IX70 fluorescence microscope (Olympus, Tokyo, Japan). DAPI and Tunnel positive cells were counted and percentage of tunnel positive cells was calculated. Scale bar on the image represents 50 µm.

SUPPLEMENTARY FIGURES

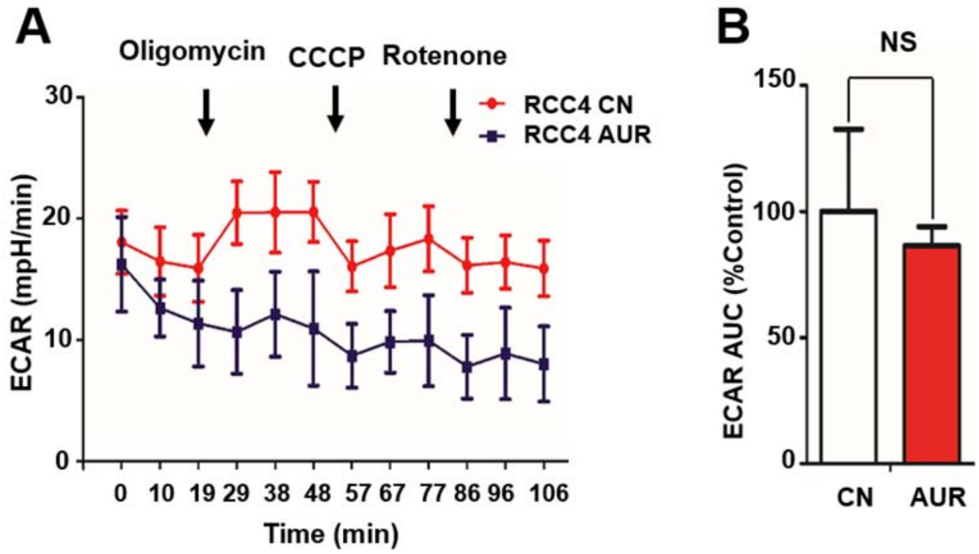

Supplementary Figure S1: Auraptene did not affect extracellular acidification rate (ECAR). A. Basal ECAR of RCC4 cells were measured by XF24 analyzer after treatment of 100 µM auraptene for 24 hours. Arrows on the graph represents the time point that oligomycin, cccp, rotenone was sequentially added. **B.** Area under curve of basal ECAR was calculated by XF24 program and represented by mean and SD. NS, no significance.

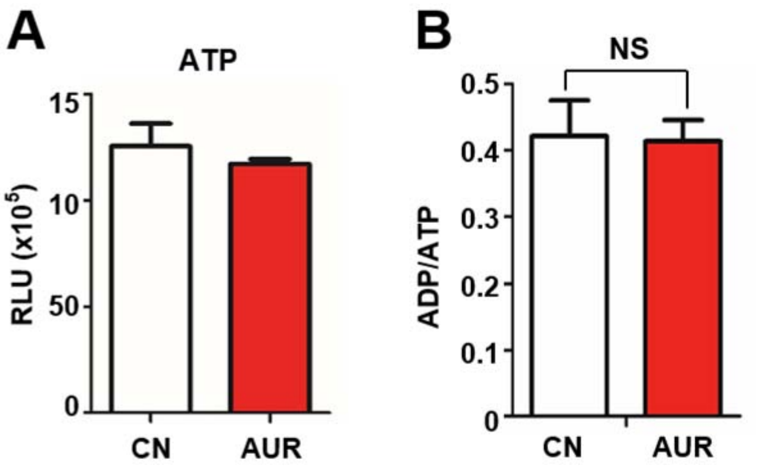

Supplementary Figure S2: Auraptene did not reduce intracellular ATP level and ADP/ATP ratio. A. Intracellular ATP level of RCC4 cells cultured in the presence or absence of 100 μM auraptene for 24 h was assessed and presented by RLU($\times 10^5$) after measured by luminometer. **B.** ADP/ATP ratio was calculated from the value of luminescence. NS, no significance.

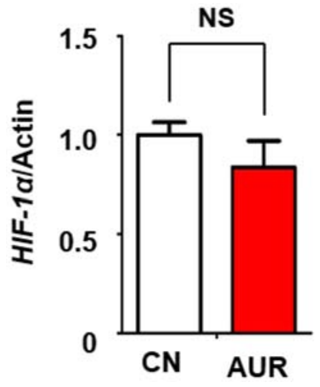

Supplementary Figure S3: Auraptene did not affect HIF-1α mRNA expression. mRNA expression of *Hif-1α* in RCC4 cells cultured in the presence or absence of 100 μM auraptene for 24 h was assessed by real-time PCR. Values are expressed as fold change. Actin was used as a control. Data represented by mean and SD. The bars on graph represent standard deviation of triplicate samples. Significant difference: ns, no significance.

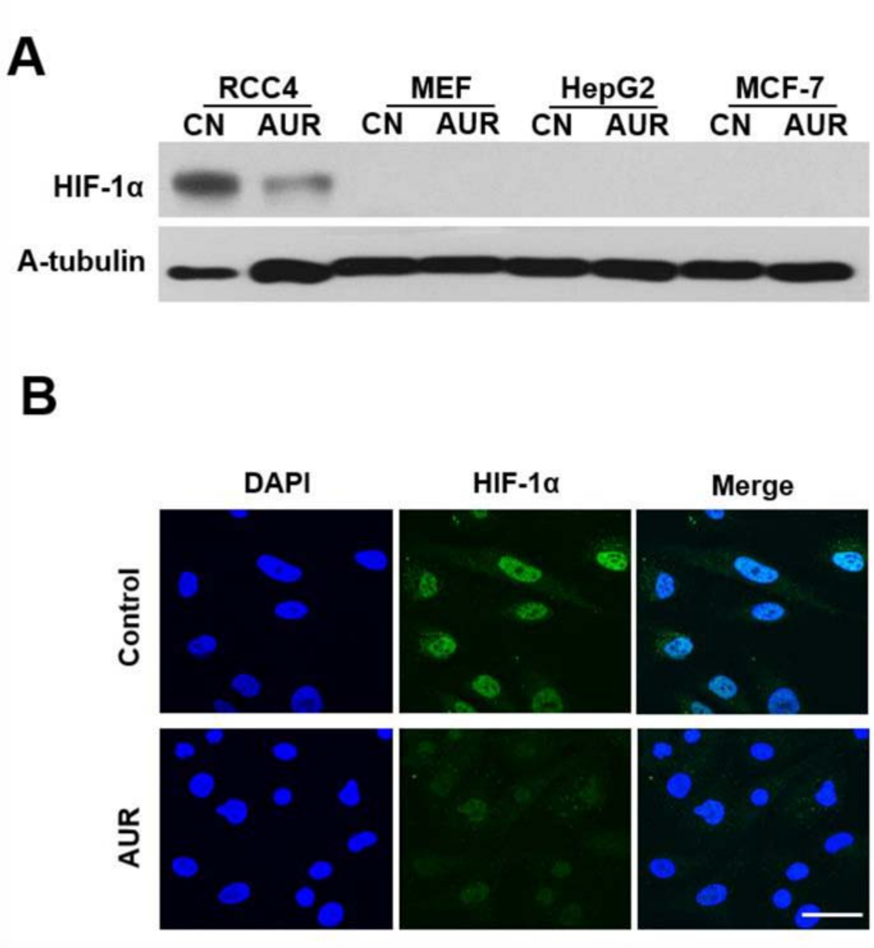

Supplementary Figure S4: Auraptene reduced HIF-1α Protein level in RCC4 cells stably expressing HIF-1α. A. RCC4, MEF, HepG2 and MCF-7 cells were cultured for 24 h in the absence or presence of 100 μM auraptene. Protein level of HIF-1α and α-tubulin were assessed by Western blotting. **B.** Confocal immunofluorescence analysis of RCC4 cells treated with either 100 μM auraptene (bottom) or DMSO (upper) using HIF-1α Ab (green). Nucleus was stained with DAPI (blue). Merged images are placed on right pannel. Magnification: x600. Scale bar in G is 50 μm.

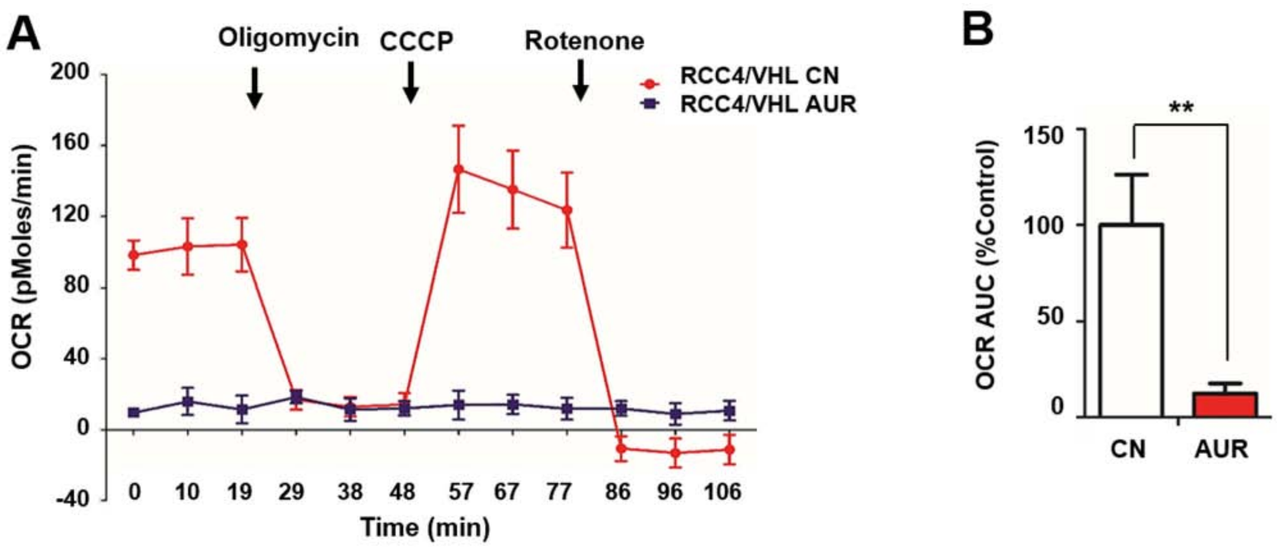

Supplementary Figure S5: Auraptene reduced oxygen consumption rate of RCC4/VHL. A. Oxygen consumption rate of RCC4/VHL cells were measured by XF24 analyzer after incubation in DMSO or 100 μM auraptene for 24 hours. Arrows marked on the graph indicate the time points of mitochondrial inhibitors administration. 2 μg/ml oligomycin, 5 μM cccp and 2 μM rotenone were sequentially added to media. **B.** Area under curve of basal OCR was calculated by XF24 software. Data represented by mean and SD. The bars on graph represent standard deviation of triplicate samples. Significant difference: $^{**}P < 0.01$.

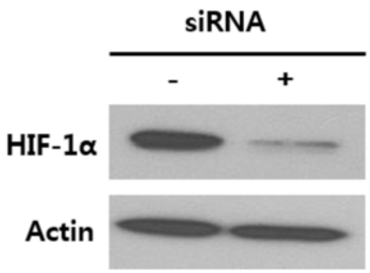

Supplementary Figure S6: siRNA transfection showed reduction of HIF-1α in RCC4 cells. 48 hours of siHIF-1α transfection greatly decreased HIF-1α protein in RCC4 cells. Protein level of HIF-1α and actin were detected by western blotting.

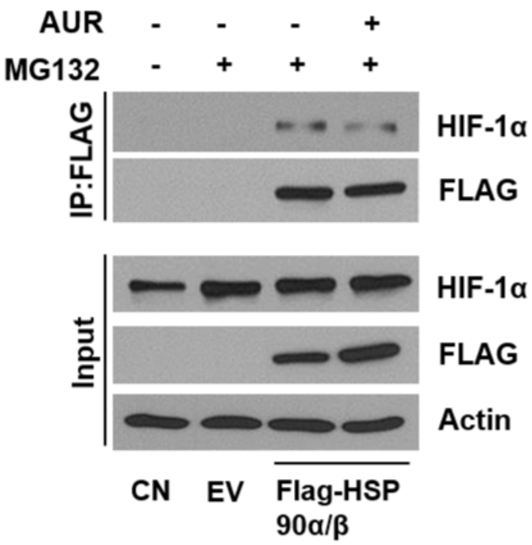

Supplementary Figure S7: Auraptene did not affect HIF-1α and HSP90 association. RCC4 cells were transfected with FLAG-HSP90 plasmid and treated with DMSO or auraptene. Immunoprecipitation assay was used to detect HIF-1α and HSP90 association. Protein level of FLAG, HSP90, HIF-1α and actin were detected by western blotting.

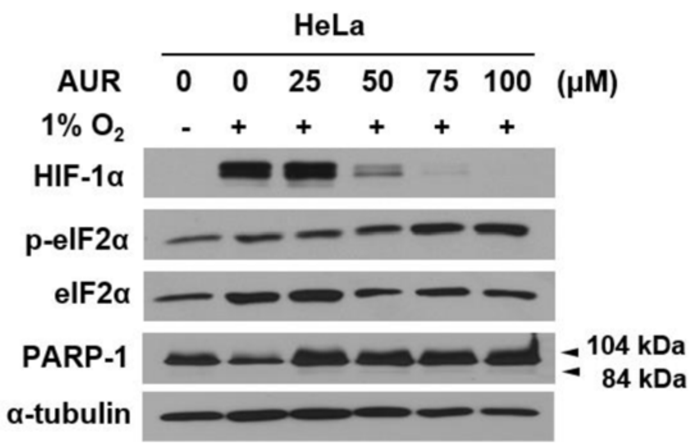

Supplementary Figure S8: Auraptene reduced HIF-1α protein under hypoxic condition in HeLa cells and induced eIF2α phosphorylation. HeLa cells were treated with DMSO or 100 µM auraptene and cultured under normoxia and hypoxia condition for 24 h. Protein levels of HIF-1α, total eIF2α, p-eIF2α, PARP-1 and α-tubulin were detected by western blotting.

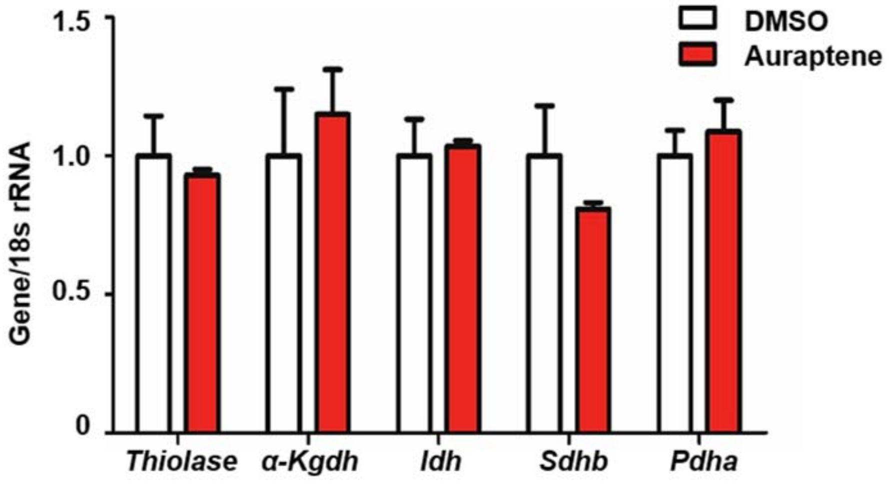

Supplementary Figure S9: Auraptene did not affect beta oxidation related gene and TCA cycle related gene. mRNA expression of *Thiolase, α-Kgdh, Idh, Sdhb* and *Pdha* in RCC4 cells cultured in the presence of DMSO or 100 µM auraptene for 24 h was assessed by qPCR. Values are expressed as relative expression. 18s rRNA was used as a control. Data are presented as mean and SEM (bars) of triplicate samples.

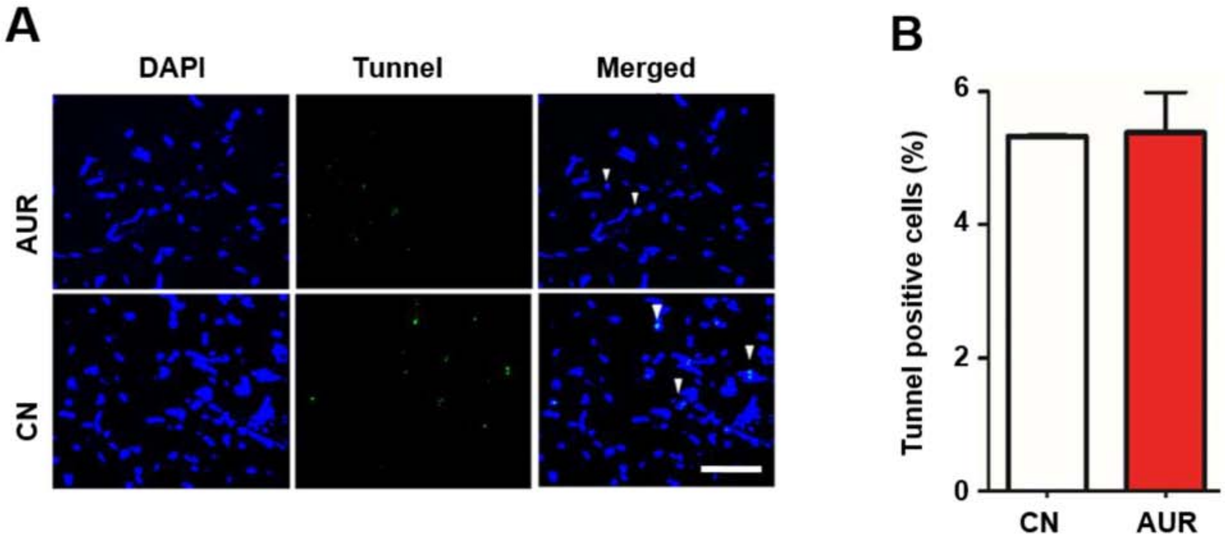

Supplementary Figure S10: Auraptene did not alter the Tunnel positive cells *in vivo*. A. Tumors detached from xenograft mouse which has been intratumorally injected with DMSO or auraptene were stained with a marker of apoptosis by Tunnel assay (n = 10, each group). Tunnel positive cells detected by green fluorescence and DAPI were displayed together. White arrows indicate the cells positive for both Tunnel and DAPI. Scale bar on the image represents 50 μm. **B.** the number of DAPI or Tunnel positive cells were counted and percentage of Tunnel positive cells were represented by graph. NS, no significance.

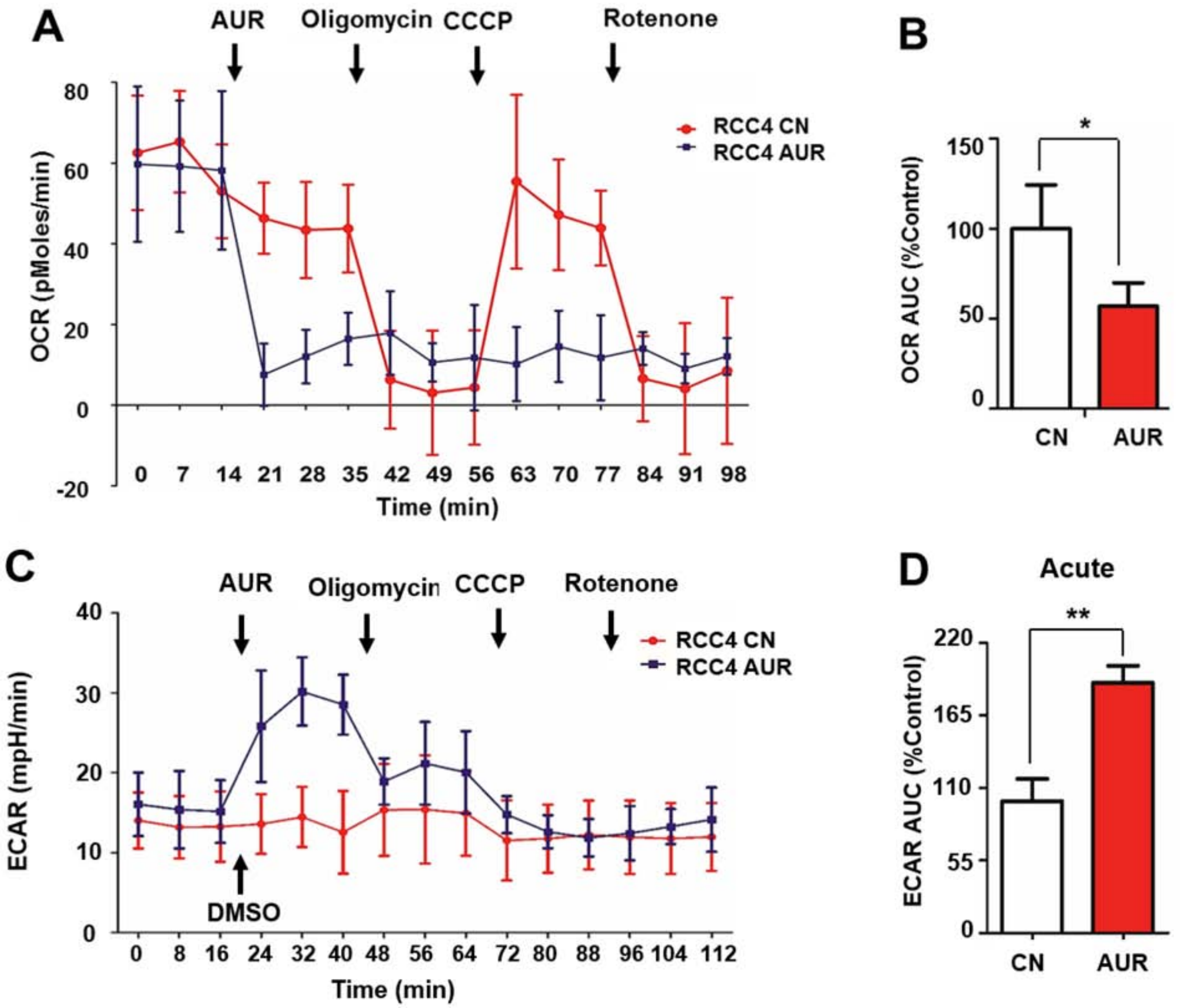

Supplementary Figure S11: Auraptene acutely reduced OCR and increased ECAR of RCC4. A, C. OCR of RCC4 cells or ECAR were measured by XF24 analyzer after adding DMSO or 200 μM auraptene. Arrows marked on the graph indicate the time points of mitochondrial inhibitors administration. 2 μg/ml oligomycin, 5 μM cccp and 2 μM rotenone were sequentially added to media. **B, D.** Area under curve of basal OCR or ECAR was calculated by XF24 software. Data represented by mean and SD. The bars on graph represent standard deviation of triplicate samples. Significant difference: *$P < 0.05$.
